# Safety and Efficacy of a Novel Sealant‐Based Vascular Closure Device Following Electrophysiology Procedures: ReliaSeal Trial

**DOI:** 10.1111/jce.16623

**Published:** 2025-03-17

**Authors:** John Summers, Vijay Swarup, Ian Parker, Joseph Bumgarner, Andrew Brenyo, Layth Saleh, Saihari Sadanandan, Javier Sanchez, Robert Beasley, Sanjaya Gupta, other members of the ReliaSeal Study Group, Muhammad Akram Khan, Mario Pascual, Norman Ramirez, Nusrath Sultana, George Adams

**Affiliations:** ^1^ South Oklahoma Heart Research Center Oklahoma City Oklahoma USA; ^2^ Arizona Heart Rhythm Center Phoenix Arizona USA; ^3^ Oklahoma Heart Hospital South Oklahoma City Oklahoma USA; ^4^ NC Heart and Vascular Research Raleigh North Carolina USA; ^5^ Piedmont Medical Center & OnSite Clinical Solutions Rock Hill South Carolina USA; ^6^ St. Anthony's Hospital, Lakewood, and St. Anthony North Hospital Westminster Colorado USA; ^7^ Colorado Heart and Vascular PC Lakewood Colorado USA; ^8^ St. Joseph's Hospital and Tampa Cardiovascular Interventions and Research Tampa Florida USA; ^9^ Medical City Dallas Heart Hospital Dallas Texas USA; ^10^ Palm Vascular Centers Research Ft. Lauderdale Florida USA; ^11^ Saint Luke's Hospital of Kansas City Kansas City Missouri USA

**Keywords:** atrial fibrillation, catheter ablation, duplex ultrasound, early ambulation, vascular closure device, venous closure

## Abstract

**Background:**

While manual compression (MC) remains the standard of care to achieve hemostasis, a novel vascular closure device (VCD) was designed to achieve faster hemostasis without compromising safety. The objective of the ReliaSeal trial was to evaluate the safety and effectiveness of the Mynx Control Venous VCD to close a single or multiple femoral venous access sites in one or both limbs in patients undergoing catheter‐based procedures.

**Methods:**

The ReliaSeal trial was a prospective, randomized controlled trial comparing VCD to MC in patients undergoing catheter‐based procedures requiring single or multiple access sites utilizing up to 12F sheaths. Endpoints included time to ambulation (TTA), time to hemostasis (TTH), time to discharge eligibility (TTDE), procedural/device success, and 30‐day major and minor access site complications.

**Results:**

Two hundred and seventy subjects were randomized to the VCD or MC arm (*n* = 177 vs. *n* = 93) with a mean age of 66.7 ± 11.27 years. TTA, TTH, and TTDE were significantly reduced in VCD subjects compared to MC (TTA: 2.6 ± 1.03 vs. 5.1 ± 4.35 h, *p* < 0.001; TTH: 2.1 ± 1.79 vs. 11.4 ± 7.19 min, *p* < 0.001; TTDE: 3.1 ± 1.24 vs. 5.5 ± 4.58 h, *p* < 0.001, respectively). Procedural and device success was achieved in 100% of VCD subjects, compared to 98.9% of the MC group. No major or minor complications occurred in the VCD group; with a 5% minor complication rate in the MC group.

**Conclusion:**

The use of VCD resulted in significant reductions in TTA, TTH, and TTDE, with no major or minor complications and a high success rate.

## Summary

- **What is known:**
  - Manual compression (MC) remains the standard of care to achieve hemostasis following catheter ablation for atrial fibrillation at many hospitals.
  - There is a growing need to achieve efficient, safe, and effective post‐procedure venous hemostasis in order to improve the post‐procedure recovery process to reduce vascular access complications, reduce patient discomfort, and shorten the recovery period to facilitate same‐day discharge.
- **What the study adds:**
  - The ReliaSeal trial was a prospective, randomized controlled trial comparing vascular closure devices (VCD) to manual compression (MC) in patients undergoing catheter‐based procedures requiring single or multiple access sites utilizing up to 12F sheaths.
  - Compared to subjects receiving MC, there was a shorter time to ambulation (TTA), time to hemostasis (TTH), and time to discharge eligibility (TTDE) in subjects receiving VCD.
  - VCD use was associated with a high success rate with no major or minor complications.

## Graphical abstract

In a clinical trial of a novel sealant‐based VCD to achieve hemostasis following endovascular procedures, patients randomized to receive the MYNX CONTROL VENOUS VCD had decreased TTH, TTA, and TDE, as compared to patients who received MC.

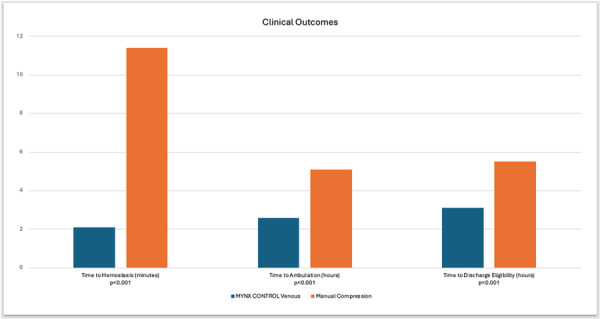

**Abbreviations**
CEC: Clinical Event Committee
DSMB: data safety and monitoring board
DUS: duplex ultrasound
DVT: deep venous thrombosis
ITT: intent‐to‐treat
MC: manual compression
PEG: polyethylene glycol
TTA: time to ambulation
TTDE: time to discharge eligibility
TTH: time to hemostasis
VCD: vascular closure device,

## 1 Introduction

There have been tremendous advances in the field of cardiac electrophysiology and catheter ablation of arrhythmias that have led to an increase in procedural volume worldwide [1, 2]. This growth has been facilitated by improvements in ablation technology and techniques that have improved the efficiency and safety of these procedures. This, in turn, led to a need to improve the postprocedure recovery process to reduce vascular access complications, reduce patient discomfort, and shorten the recovery period to facilitate same‐day discharge. Consequently, the need for techniques to achieve efficient, safe, and effective postprocedure venous hemostasis has grown also. Initially, the figure of eight subcutaneous suture was used as an alternative to manual compression (MC) to achieve hemostasis after electrophysiology procedures [3, 4]. However, it was observed that the figure of eight suture was still prone to bleeding complications after certain electrophysiology procedures [5]. This led to the development of venous VCDs for electrophysiology procedures. Several venous VCDs have been developed and studied in electrophysiology procedures, including suture‐based devices [6, 7, 8] and collagen plug‐based devices [9, 10, 11].

**Figure 1:**
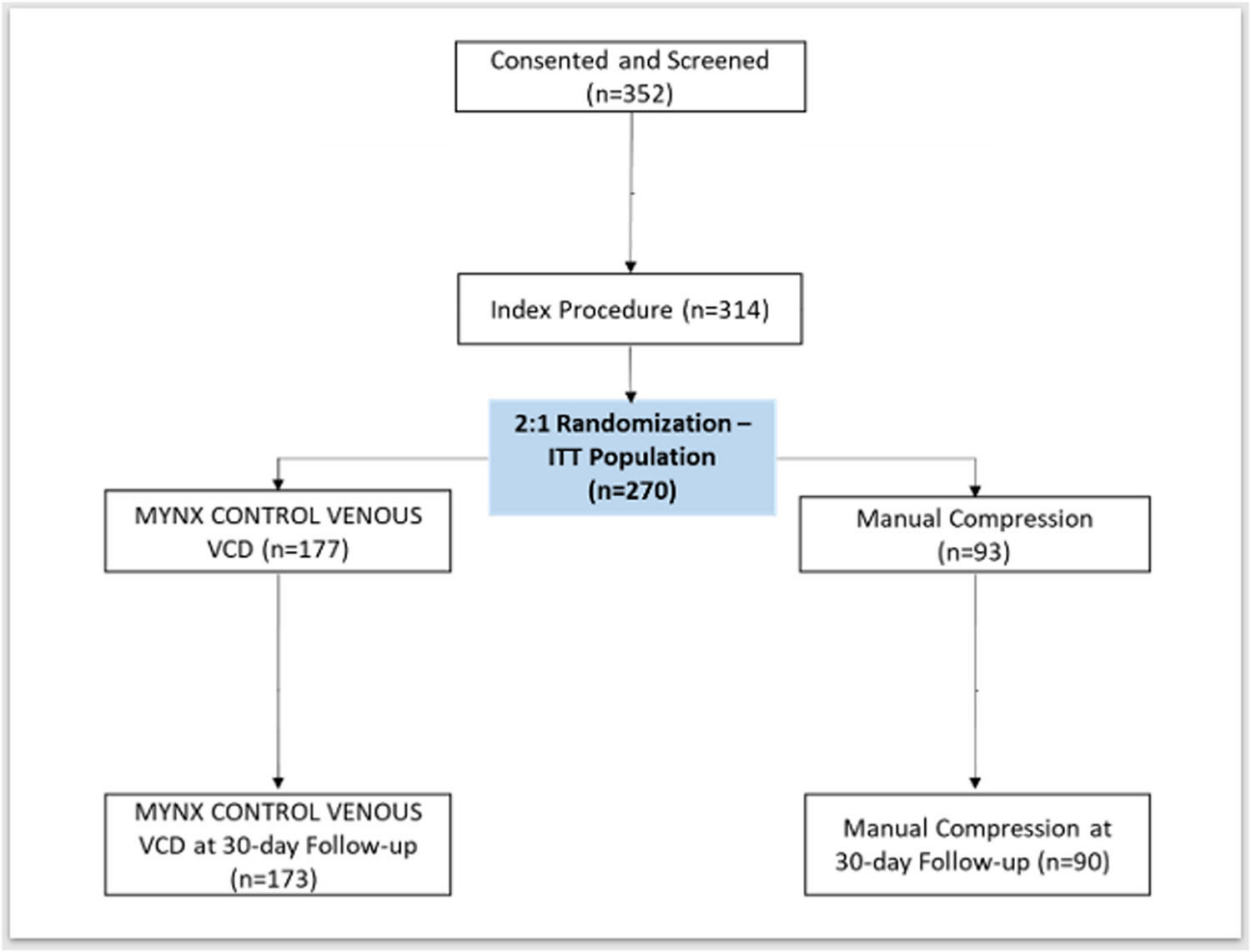
Subject disposition.

**Figure 2:**
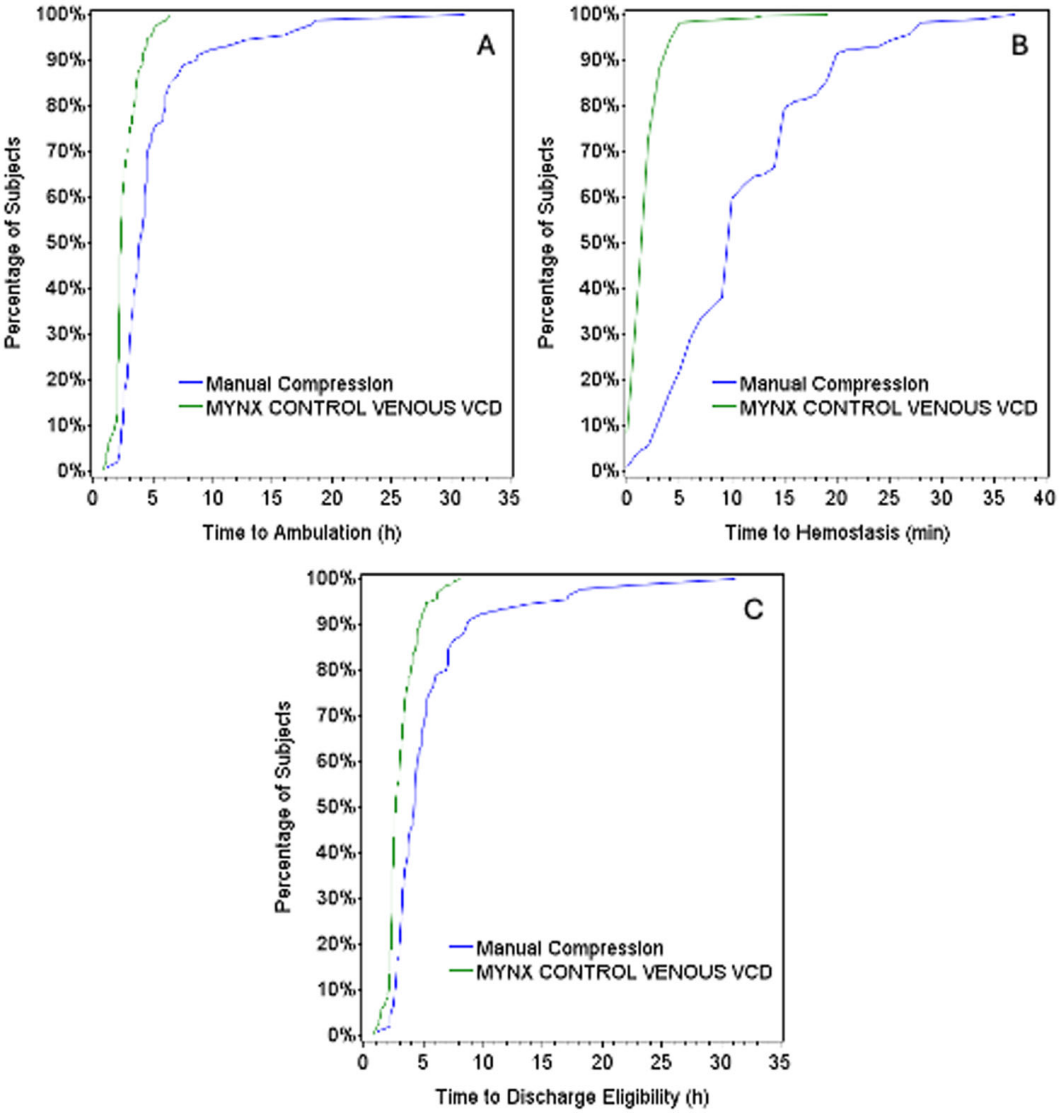
Time course for MCV VCD and MC for TTA (A), TTH (B), and TTDE (C).

In this study, we utilized the Mynx Control Venous Vascular Closure Device (MCV VCD), which is a single‐use VCD designed to achieve hemostasis for 6F‐12F (inner diameter) access sites (Supporting Information S1: Figure 1). Sheaths with an outer diameter of up to 15Fr were used in this trial. The device is inserted through an existing sheath and a balloon is inflated in the vessel. The device and sheath are retracted, and the balloon is used to provide temporary hemostasis (Video 1). A tension indicator on the device handle provides information on the appropriate amount of traction to achieve hemostasis without dislodging the device from the access site. Hemostasis is then achieved by pressing a button on the device to deliver a water‐soluble, resorbable, extravascular polyethylene glycol (PEG) hydrogel that rapidly expands inside the tissue tract by absorbing blood and subcutaneous fluids. This provides an immediate seal of the access site and facilitates natural hemostasis in about 2 min. The balloon is then deflated, the device is removed, and brief manual pressure is applied to allow the sealant to expand to fill the lumen left by the device and achieve final hemostasis (Supporting Information S1: Figure 2). There is no residual endovascular material, and the PEG sealant is fully absorbed within 30 days. If necessary, reintervention in the same vessel is possible even within the first 30 days. This device is based on the MYNX VCD Product Family and the Exoseal VCD Product Family, which provide hemostasis at femoral access sites in patients who have undergone endovascular procedures utilizing a 5F, 6F, or 7F procedural sheath. For this study, we expanded the indication of this device to include up to 12F (inner diameter) procedural sheaths for femoral venous access. Procedures using this larger bore sheath include but are not limited to, cardiac ablations, structural heart procedures, and cardiac interventions. The goal of this study was to evaluate the safety and efficacy of the MCV VCD compared to MC after catheter‐based procedures via the common femoral vein(s). We evaluated time to hemostasis (TTH), time to ambulation (TTA), and the rate of access site closure‐related complications, as well as several secondary endpoints including time to discharge eligibility (TTDE).

## 2 Materials and Methods

The ReliaSeal trial (NCT05554471) was a multicenter, prospective, randomized, controlled, open‐label trial designed to evaluate the safety and efficacy of the MCV VCD versus MC in patients undergoing catheter‐based endovascular procedures, such as cardiac ablation, using procedural sheaths 6F‐12F (inner diameter) in size. Sheaths up to 15Fr outer diameter were used in this trial.

The trial included 270 adult patients ( ≥ 18 years of age) randomized to either the device arm or the MC arm using a 2:1 randomization scheme (177 MCV VCD patients and 93 MC patients). Two roll‐in patients per investigator were allowed. Included patients were those scheduled to undergo catheter‐based procedures via the common femoral vein(s) using 6F‐12F introducer sheaths after signing an informed consent form approved by the Institutional Review Board at each enrolling site. Exclusion criteria included vascular surgery or repair in the vicinity of the target access site within the previous 90 days, active systemic or cutaneous infection in the vicinity of the target site, history of deep vein thrombosis, pulmonary embolism, or thrombophlebitis within 6 months of the procedure, history of bleeding disorder such as hemophilia or von Willebrand's disease, use of systemic steroids within 30 days of the procedure, presence of thrombocytopenia (platelet count < 100 000 cells/mm^3^) or anemia (hemoglobin < 10 g/dL, hematocrit < 30%), and patients with a life expectancy of less than 30 days.

During the primary endovascular procedure, patients who met additional exclusion criteria could further be excluded from the trial. These criteria included a venous access site location above the inguinal ligament, an attempt at femoral arterial access, index procedural complication which might interfere with routine recovery, ambulation, or discharge eligibility, or intra‐procedural bleeding around the sheath, suspected intraluminal thrombus, hematoma, pseudoaneurysm, or AV fistula.

All patients who were randomized to either group were followed through 30 days post‐discharge. A sub‐study of this pivotal trial included noninvasive duplex ultrasound (DUS) imaging in 48 device patients and 24 MC patients predischarge, as well as at the 30‐day follow‐up visit if a complication was observed on the predischarge ultrasound. All ultrasound imaging was interpreted by an independent core lab. Additional informed consent was obtained from all patients who participated in the sub‐study.

### 2.1 Procedure

#### 2.1.1 Device Arm

All patients randomized to the device arm received the device through venous access sites with sheaths 6F to 12F (inner diameter) in size. After insertion of the catheter tip of the MCV VCD through the sheath valve, the balloon was inflated and positioned so it was abutting the venotomy site. The device was then properly positioned to deliver the sealant. The GRIP TECHNOLOGY sealant, an extravascular, water‐soluble synthetic hydrogel made of a PEG material, was then delivered, followed by a 2‐min pause to allow the sealant to actively absorb body fluid and swell within the tissue tract. While maintaining fingertip compression on the skin, the device and sheath were then retracted from the patient. The TTH was recorded for each access site and confirmed 5 min after to be maintained. This process was repeated for each of the venous access sites.

#### 2.1.2 Manual Compression Arm

MC was utilized to obtain hemostasis in the control arm following standard institutional protocols. If protamine was administered, the Investigator followed institutional practice guidelines for re‐checking ACT levels and/or waiting a specified time or for a target ACT value before removal of sheaths and application of MC. The TTH was recorded for each access site and confirmed at 5 min later to be maintained.

In both arms, periprocedural anticoagulation followed institutional protocols. All anticoagulant and antiplatelet medications were recorded on the patients' case report forms. Additionally, patients underwent a pain assessment at the time of discharge eligibility.

### 2.2 Study Endpoints

The trial included two co‐primary efficacy endpoints, TTH and TTA. TTH was defined as the elapsed time between removal of each VCD (device arm) or of the final sheath (manual compression arm) and the first confirmed venous hemostasis for each access site. TTA was defined as the elapsed time between removal of the final VCD (device group) or of the final sheath (control group) and when the patient ambulated 20 feet without evidence of rebleeding from any femoral venous access site. While patients in the MC arm ambulated per institutional guidelines, the protocol mandated that ambulation be attempted within 2–2.5 h following final VCD removal in the device arm. It was assumed that most institutions used a protocol of 5–6 h of bed rest following an ablation procedure, allowing for variability in this duration on an individual patient basis, according to clinical judgment.

Secondary efficacy endpoints included procedural success (attainment of final hemostasis at all access sites without major access site complications through 30 days), device success (ability to successfully deploy the VCD system, deliver the sealant, and achieve hemostasis in the device arm), and TTDE. TTDE was defined as the elapsed time between removal of the final VCD (device arm) or removal of the final sheath (control arm) and when the subject was eligible for discharge from the institution based on the assessment of the attending physician.

The primary safety endpoint was defined as the rate of combined major venous access site closure‐related complications through 30 days postprocedure, attributed directly to either the VCD or MC without other likely causes. The composite safety endpoint included access site‐related bleeding requiring transfusion, surgical intervention, or rehospitalization, vascular injury requiring surgical repair, access site‐related infection confirmed by culture and requiring intravenous antibiotics and/or extended hospitalization, new onset access site‐related nerve injury, and pulmonary embolism confirmed by angiography with or without surgical or endovascular intervention.

The secondary safety endpoint was the rate of minor venous access site closure‐related complications within 30 days postprocedure, attributed directly to either the VCD or MC without other likely cause.

#### 2.2.1 Endpoint Adjudication

All serious adverse events, device‐related complications and/or malfunctions, and events related to the primary safety endpoint were adjudicated by a Clinical Events Committee (CEC), which was comprised of a group of independent non‐investigator physicians. The process of adjudication of study events is detailed in the CEC charter and minimized any potential bias regarding device or procedure relatedness of those events.

The ReliaSeal trial also utilized a Data and Safety Monitoring Board (DSMB). The DSMB is an independent body of professionals comprised of physicians, a biostatistician, and/or a medical ethicist who review overall study data and/or event accrual to assess progress and identify any safety concerns or other issues.

### 2.3 Sample Size and Statistical Analyses

The average time to hemostasis (TTH) was compared between treatment groups using a generalized linear mixed model where the subject is considered a random effect with multiple access sites. TTH in the MC group was reduced by 5 min to account for the hypothesis that the time to hemostasis for the VCD group was at least 5 min less than MC. Using estimates of population means and standard deviations from historical studies, a sample size of 150 subjects (100 VCD subjects and 50 MC subjects) provided approximately 95% power to detect a difference in hemostasis times of seven [7] minutes between both groups.

For TTA, it was assumed that the VCD treatment group ambulates in 3.0 h on average and the control group ambulates in 6.0 h, and both groups have a standard deviation of 6.0 h. An effective sample size of 192 subjects (128 VCD subjects and 64 manual compression subjects) provided 90% power to detect the difference in TTA between the two groups of subjects using a Type I error of 0.025 for a one‐sided test. Adjusting for a missing data rate of 5%, a total of 204 subjects were required for the TTA co‐primary endpoint.

## 3 Results

### 3.1 Demographics and Patient Characteristics

A total of 352 subjects were screened and consented at 13 sites in the USA and, of those 352, 314 subjects met the selection criteria and were enrolled, including roll‐in subjects (*N* = 44). Of those 314, 270 subjects were randomized to either the device arm (*N* = 177) or manual compression (*N* = 93). Out of 270 randomized subjects, 173 from the device arm and 90 from the manual compression arm had a 30‐day visit post‐discharge (See Figure 1). Four VCD subjects (two lost to follow‐up and two withdrawn by investigator) and three manual compression subjects (one lost to follow‐up, one withdrawn by investigator, and one death) were excluded from the 30‐day follow‐up analyses.

The demographics of both groups were similar and are summarized in Table 1. Medical history and risk factors are presented in Table 2. A majority of subjects had atrial fibrillation (54%) and half of the enrolled subjects had hypertension. The procedures performed included ablation for atrial fibrillation/flutter (both radiofrequency and cryoablation) as well as ablation for supraventricular tachycardia, premature ventricular contractions and AV node (Supporting Information S1: Table 1). Approximately 2/3 of subjects had venous access in the right leg only and 1/3 had venous access in both legs (Supporting Information S1: Figure 3). The average number of sheaths per subject was 2.7 ± 1.1 with a range from 1 to 5 sheaths. There were 71.5% of sheaths that were less than or equal to 8.5Fr in size and 28.5% of sheaths were greater than or equal to 9Fr in size (Supporting Information S1: Figure 4; Supporting Information S1: Table 2).

**Table 1:** Baseline demographics – ITT subjects.

Subject characteristics	MCV VCD (*N* = 177 subjects)	Manual compression (N = 93 subjects)	All (*N* = 270 subjects)
Age (yrs)			
N	177	93	270
Mean ± SD	66.7 ± 11.62	66.8 ± 10.63	66.7 ± 11.27
Median (IQR)	69 (61 ‐ 75)	69 (63 ‐ 73)	69 (62 ‐ 75)
Min, Max	(19 ‐ 86)	(31 ‐ 86)	(19 ‐ 86)
Sex			
Female	33.9% (60/177)	34.4% (32/93)	34.1% (92/270)
Male	66.1% (117/177)	65.6% (61/93)	65.9% (178/270)
BMI (kg/m^2^)			
N	177	93	270
Mean ± SD	30.6 ± 6.13	29.6 ± 5.18	30.3 ± 5.82
Median (IQR)	29.8 (25.4 ‐ 35.2)	28.7 (25.5 ‐ 33.1)	29.5 (25.5 ‐ 34.5)
Min, Max	(20.1 ‐ 43.2)	(21.5 ‐ 43.8)	(20.1 ‐ 43.8)
Ethnicity			
Hispanic or Latino	5.1% (9/176)	2.2% (2/93)	4.1% (11/269)
Non‐Hispanic or Latino	89.8% (158/176)	93.5% (87/93)	91.1% (245/269)
Unknown	5.1% (9/176)	4.3% (4/93)	4.8% (13/269)
Race			
American Indian or Alaska Native	0.6% (1/177)	0.0% (0/93)	0.4% (1/270)
Asian	1.1% (2/177)	1.1% (1/93)	1.1% (3/270)
Black or African American	2.3% (4/177)	2.2% (2/93)	2.2% (6/270)
White	94.4% (167/177)	95.7% (89/93)	94.8% (256/270)
Unknown	1.7% (3/177)	1.1% (1/93)	1.5% (4/270)

**Table 2:** Medical history and risk factors – ITT subjects.

Subject characteristics	MCV VCD (*N* = 177 subjects)	Manual compression (*N* = 93 subjects)	All (*N* = 270 subjects)
Hypertension	48.0% (85/177)	53.8% (50/93)	50.0% (135/270)
Atrial fibrillation	55.4% (98/177)	52.7% (49/93)	54.4% (147/270)
Diabetes	18.1% (32/177)	16.1% (15/93)	17.4% (47/270)
Dyslipidemia	33.3% (59/177)	37.6% (35/93)	34.8% (94/270)
DVT/Thrombosis	1.7% (3/177)	1.1% (1/93)	1.5% (4/270)
Atrial flutter	10.2% (18/177)	8.6% (8/93)	9.6% (26/270)
Morbid obesity	2.3% (4/177)	0.0% (0/93)	1.5% (4/270)
Peripheral vascular disease	2.8% (5/177)	3.2% (3/93)	3.0% (8/270)

### 3.2 Pre‐ and Peri‐Procedural Anticoagulant and Antiplatelet Medication

Anticoagulant and antiplatelet medications pre‐ and peri‐procedurally were used in 81.9% of device subjects and 81.7% of manual compression subjects (Table 3). Nearly half of overall subjects (48.5%) were on Apixaban/Eliquis. Activated clotting time (ACT) at the conclusion of the procedure was reported on a patient level but was not collected on all patients, as it was not a required assessment per protocol. The ACT was 315.4 ± 89.1 s (*N* = 50/177), 326.9 ± 72.2 s (*N* = 24/93), and 319.1 ± 83.7 s (*N* = 74/270) in the MCV VCD group, the MC group, and the combined cohorts, respectively. The proportion of patients that received protamine for heparin reversal in the three groups was 51.4% (90/175), 51.1% (47/92), and 51.3% (137/267), respectively (protamine administration from three patients was not collected because they were terminated from the study before the procedure).

**Table 3:** Pre‐ and peri‐procedure anticoagulant/antiplatelet medication – ITT subjects.

Medication	MCV VCD (*N* = 177 subjects)	Manual compression (*N* = 93 subjects)	All (*N* = 270 subjects)
Warfarin	1.1% (2/177)	2.2% (2/93)	1.5% (4/270)
Prasugrel	0.6% (1/177)	0.0% (0/93)	0.4% (1/270)
Pradaxa	1.1% (2/177)	0.0% (0/93)	0.7% (2/270)
Brilinta	0.6% (1/177)	0.0% (0/93)	0.4% (1/270)
Rivaroxaban/Xarelto	10.2% (18/177)	11.8% (11/93)	10.7% (29/270)
Heparin	38.4% (68/177)	40.9% (38/93)	39.3% (106/270)
Clopidogrel/Plavix	7.9% (14/177)	10.8% (10/93)	8.9% (24/270)
Aspirin/ASA	23.7% (42/177)	26.9% (25/93)	24.8% (67/270)
Apixaban/Eliquis	50.3% (89/177)	45.2% (42/93)	48.5% (131/270)
Any of the above	81.9% (145/177)	81.7% (76/93)	81.9% (221/270)

### 3.3 Technical Success

There was a total of 470 and 249 venous access closure sites in the device and manual compression intent‐to‐treat (ITT) groups, respectively. Procedural success, defined as the attainment of final hemostasis at all venous access sites without major access site closure‐related complications through 30 days, was achieved in 100.0% (171/171) of the device subjects and 98.9% (89/90) of the manual compression subjects. One MC subject experienced access site‐related bleeding requiring transfusion and surgical intervention. Six of the 177 VCD subjects and three of the 93 manual compression subjects had missing data. Device success, defined as the ability to successfully deploy the VCD delivery system, deliver the PEG hydrogel sealant, and achieve hemostasis was achieved in all ITT cases (470/470).

### 3.4 Efficacy Endpoints

The average TTH was compared between groups using a generalized linear mixed model where the subject is considered a random effect with multiple access sites.

Both study co‐primary efficacy endpoints of TTA and TTH were met (Table 4; Figure 2A,B). TTA was decreased in the VCD arm compared to the MC arm by 49% (2.6 ± 1.03 vs. 5.1 ± 4.35 h; *p* < 0.001). TTH was also decreased in the VCD arm compared to the manual compression arm by 82% (2.1 ± 1.79 vs. 11.4 ± 7.19 min; *p* < 0.001). The results support the hypothesis that TTH is at least 5 min less for subjects treated with the VCD compared to those receiving MC.

**Table 4:** Primary efficacy outcomes – ITT subjects.

Subject characteristics	MCV VCD (*N* = 177 subjects)	Manual compression (*N* = 93 Subjects)	*p*‐value
TTA (hr)			< 0.001^a^
N (# of subjects)	172	91	
Mean ± SD	2.6 ± 1.03	5.1 ± 4.35	
Median (IQR)	2.28 (2.08–3.08)	3.90 (2.97–5.15)	
Min, max	(0.83 – 6.42)	(1.15–31.17)	
TTH (min)			< 0.001^b^
N (# of access sites)	470	249	
Mean ± SD	2.1 ± 1.79	11.4 ± 7.19	
Median (IQR)	2 (1–3)	10 (6–15)	
Min, max	(0–19)	(0–37)	

^a^ The average TTA in each treatment group was compared using a Wilcoxon rank sum test.

^b^ The average TTH was compared between the treatment groups using a generalized linear mixed model where subject is considered a random effect with multiple access sites.

The secondary efficacy endpoint of TTDE was also met. On average, TTDE was reduced by 44% in the VCD arm compared to the manual compression arm (3.1 ± 1.24 vs. 5.5 ± 4.58 h; *p* < 0.001) (Table 5; Figure 2C).

**Table 5:** Time to discharge eligibility (in hours)– ITT subjects.

Subject characteristics	MCV VCD (*N* = 177 subjects)	Manual compression (*N* = 93 subjects)	Difference [95% CI]	*p*‐value a
TTDE (hr)				< 0.001
N	173	91		
Mean ± SD	3.1 ± 1.24	5.5 ± 4.58	–2.4 [–3.4, –1.4]	
Median (IQR)	2.67 (2.35 –3.52)	4.25 (3.12 –5.67)		
Min, Max	(0.83 –8.05)	(1.15 –31.17)		

^a^ The average TTDE was compared using a Wilcoxon rank sum test.

### 3.5 Safety Endpoints

There were no major venous access site closure‐related complications in the VCD arm through 30 days of follow‐up. In the manual compression arm, one subject experienced access site bleeding on the day of the procedure. Following close monitoring in the ICU, the subject was released 3 days following the procedure in stable condition. The primary safety endpoint of non‐inferiority was met with the upper limit of the one‐sided 97.5% confidence interval bound of 0.9%, which was less than the 5% non‐inferiority window (Table 6).

**Table 6:** Primary safety outcomes – ITT subjects.

Subject Characteristics	MCV VCD (N = 177 Subjects)^a^	Manual Compression (N = 93 Subjects)^a^	Difference [95% CI]^b^
Major complications of the target limb access site within 30 Days	0.0% (0/229)	0.8% (1/119)	−0.8%
	[0.0%, 1.6%]	[0.0%, 4.6%]	[−4.6%, 0.9%]

^a^ Results are reported as: percentage of limbs with major complications (number of limbs with major complications/total number of evaluable limbs) [confidence interval]. 95% CI is from the Clopper–Pearson method. Total number of evaluable limbs = treated limbs that had last contact date >= 23 days or had major complications before 30 days.

^b^ 95% confidence interval used the method of variance estimates recovery (MOVER).

Similarly, there were no minor venous access site closure‐related complications through 30 days of follow‐up in the VCD group that were related to the device. There were six minor complications in the manual compression group. Details of those events are shown in Table 7.

**Table 7:** Minor complications within 30 Days – ITT subjects.

	MCV VCD (*N* = 177 subjects)	Manual compression (*N* = 93 subjects)	All (*N* = 270 subjects)
Number of limbs treated	236	122	358
Minor complications of target limb access site within 30 days	0.0% (0/229)	5.0% (6/119)	1.7% (6/348)
Pseudoaneurysm not requiring treatment	0.0% (0/229)	0.8% (1/119)	0.3% (1/348)
Access site‐related hematoma > 6 cm documented by ultrasound	0.0% (0/229)	0.8% (1/119)	0.3% (1/348)
Access site‐related bleeding requiring > 30 min to achieve hemostasis	0.0% (0/229)	0.8% (1/119)	0.3% (1/348)
Late access site‐related bleeding (following hospital discharge eligibility)	0.0% (0/229)	1.7% (2/119)	0.6% (2/348)
Ecchymosis	0.0% (0/229)	0.8% (1/119)	0.3% (1/348)

### 3.6 Patient Experience

The protocol included an assessment of pain at the access site(s) at the time of discharge eligibility, measured by pain scores (scale of 1 to 10, 10 being the highest). More than 82% of subjects in the VCD group rated their pain level at 0 or 1, compared to 71.9% in the manual compression group (Supporting Information S1: Table 3).

### 3.7 Ultrasound Sub‐Study

A total of 47 subjects in the device arm and 25 subjects in the manual compression arm participated in the DUS sub‐study. The subjects in both groups were required to undergo a DUS before discharge and, if any complications were noted at discharge, those subjects were required to undergo a DUS at their 30‐day follow‐up visit.

In the device arm, five subjects had findings of intraluminal thrombus (*N* = 3) and hematoma < 6 cm (*N* = 3) at discharge. One of these subjects had a finding of both intraluminal thrombus and hematoma < 6 cm at discharge. Two of the five subjects were lost to follow‐up before the 30‐day visit. The other three subjects had no findings on the 30‐day DUS. In the manual compression arm, one subject had findings of hematoma < 6 cm and pseudoaneurysm at discharge and there were no findings at the 30‐day visit. The rate of these subclinical complications detected by ultrasound was very low and not significantly different between the two arms (0.1% in the device arm vs 0.4% in the control arm, P = NS).

## 4 Discussion

The multicenter, prospective, randomized, controlled ReliaSeal trial evaluated the safety and efficacy of the MCV VCD versus MC in patients undergoing endovascular procedures. The results indicate a statistically significant decrease in the mean TTH of 9.3 min and a decrease in the mean TTA of 2.5 h in patients who received the VCD as compared to MC. The ReliaSeal trial also demonstrated a significant reduction in the mean TTDE of 2.4 h in patients who received the VCD as compared to MC. Importantly, there was 100% successful device deployment and there were no minor or major complications in the VCD group.

It is important to place the results into the context of other published VCD clinical trials. While no published trials compared two VCDs directly, most of the published VCD trials were compared to MC, which allows indirect comparison. The AMBULATE trial, which evaluated the efficacy and safety of the VASCADE MVP device, reported a median TTH of 5.1 min and a median TTA of 2.2 h as compared to the median TTH of 2 min and median TTA of 2.28 h reported in the ReliaSeal trial [9]. In comparison, the most recent trial featuring a suture‐mediated closure (SMC) device reported a median TTH of 6.5 min and a median TTA of 3.2 h [8]. The median TTDE was 2.67 in the ReliaSeal trial as compared to 2.5 h in the AMBULATE trial [9]. This same endpoint was not evaluated in SMC trials but the most recent clinical trials reported a median time to discharge of 5.0 and 5.95 h [7, 8]. The procedural success rate for VCD deployment was 100% in the ReliaSeal trial, 98% in the AMBULATE trial, and 99.2% in the most recent SMC trial [8, 9]. While direct comparison is not possible between these studies, the results of the ReliaSeal clinical trial demonstrate comparable efficacy to other published VCD clinical trials.

In terms of safety, the AMBULATE trial reported no major complications, 2 (1% rate) minor complications and 15 (7.5% rate) closure‐related adverse events, the most common of which was rebleeding after initial hemostasis was confirmed for 5 min [9]. The most recent SMC study reported no major complications and 6 (16.7% rate) minor complications; however, this included complications detected by DUS only. In comparison, the ReliaSeal trial reported no major or minor complications (0% rate) as evidenced by DUS at the 30‐day follow‐up visit and demonstrated the lowest reported incidence of major and minor access site‐related complications in the published literature.

It is also relevant to put the ReliaSeal trial in context with the figure of eight suture clinical trials. The early trials evaluated the safety and efficacy of figure of eight sutures with a standard protocol of 3–4 h of bed rest post‐sheath removal [3, 4]. While these trials suggested the figure of eight suture was safe and effective, other figure of eight suture trials demonstrated an 11% bleeding complication rate, which was associated with large bore (15F) access used in Cryoballoon ablation [5]. The ReliaSeal trial reported a shorter median TTA and a lower rate of complications than reported for figure of eight suture in these studies. The only trial that directly compared a figure of eight suture with VCD and MC was the VACCAR trial, which demonstrated a median TTH of 7 min for VCD, 9 min for figure of 8, and 20 min for MC (*p* < 0.001). Median TTA was 2.2 h for VCD and figure of 8 versus 6.5 h for MC (*p* < 0.01). There was no significant difference in major or minor complications between the three groups [6]. Compared to this trial, the ReliaSeal study reported a shorter median TTH and a comparable rate of TTA and complication rate. The figure of eight technique is fundamentally different from VCD in that it does not close the vessel itself, rather it closes subcutaneous tissue above the access site, thereby providing indirect pressure to achieve hemostasis. In the experience of several of the authors, the figure of eight stitches can cause access site pain for patients and, unlike VCDs, it requires removal of the stitch hours after the procedure. The removal of the stitch can cause pain to the patient and can be disruptive to workflow.

There are several technical aspects of the MCV VCD that can explain the efficacy and safety results demonstrated in the ReliaSeal trial. The endovascular balloon and the tension indicator on the MCV VCD provide a reliable indicator of hemostasis. The MCV VCD is the only closure device designed with an ergonomic handle and one‐button deployment, resulting in ease of use. As opposed to suture‐mediated closure devices, there is no permanent endovascular material with the MCV VCD, which may explain the differences in rates of thrombosis and deep venous thrombosis seen in the DUS sub‐study of the SMC device [8]. Another differentiator of the MCV VCD is the sealant itself. PEG, is a hydrogel which absorbs blood and interstitial fluid with resultant expansion three to four times its original size, thereby achieving rapid hemostasis. Breakdown and absorption occur through hydrolysis within 30 days, leaving nothing behind, as compared to 90 days of absorption with collagen‐based VCDs [12]. PEG has excellent biocompatibility, excellent tissue adhesion and mechanical compliance, and negligible immunogenicity. It is not associated with initiation of inflammatory processes or exaggerated scar formation, nor is it thrombotic, having no effect on initiation of the coagulation cascade [13, 14].

The ReliaSeal clinical trial utilized a robust clinical design: a multi‐center, randomized, controlled prospective, open‐label study with relevant endpoints of TTH, TTA, and TTDE. The study was adequately powered to detect a significant difference in these endpoints. However, like any major clinical trial, there were limitations of this study. First, there was no standardized protocol for TTA and this was left to institutional protocol. However, this was standard for all other VCD trials that used TTA as an endpoint. Therefore, it is important to realize that this trial demonstrated the feasibility of a shorter TTA with VCD, but it is not known if a shorter TTA was possible with MC as that hypothesis was not tested. Second, this was an unblinded study where all care providers knew the treatment assignment. Third, the ReliaSeal trial did not compare the MCV VCD to other VCDs or figure of 8, making direct comparisons of safety and efficacy challenging. However, all other VCD trials used manual compression as a control group and ReliaSeal was consistent with these other studies in this regard and makes indirect comparisons among these studies possible through utilization of a common control group. Finally, when the ReliaSeal trial was conceived, the maximum sheath used for catheter ablation was 15Fr (outer diameter). However, with the advent of pulsed‐field ablation that occurred after the ReliaSeal trial concluded, it is common to use 17Fr (outer diameter) sheath sizes now. This sheath size was not studied in the ReliaSeal study so it is not known how the MCV VCD device would perform with a larger sheath.

## 5 Conclusion

The ReliaSeal trial demonstrated the safety and efficacy of the MCV VCD as compared to MC in patients undergoing endovascular procedures using procedural sheaths 6F‐12F in size. By demonstrating significant reductions in TTH, TTA, and TTDE, the ReliaSeal trial demonstrated superiority of the MCV VCD over MC and allowed indirect comparisons to other published VCD trials.

### 5.1 ReliaSeal Study Group Additional Members

Muhammad Akram Khan, MD, Mario Pascual, MD, Norman Ramirez, MD, Nusrath Sultana, MD MBA, George Adams, MD.

**Video 1:** Ultrasound image of Mynx Venous Vascular Closure Device in femoral vein. In the first clip, the vein is seen in cross‐section and the balloon at the tip of the device shaft is shown moving to the vessel surface, which provides hemostasis and also facilitates sealant deposit on the venotomy surface and not within the vessel. In the second clip, the device is being retracted to trigger the deployment mechanism to allow sealant deposition. This clip shows tenting of the vessel and surrounding tissue indicating that the balloon remains in the vessel as manual traction is applied.

## Conflicts of Interest

Dr. Summers consults for Cordis. Dr. Bumgarner consults for Cordis. Dr. Gupta consults for Boston Scientific, Abbott, Cordis, and Respicardia.

## Supporting information

Supporting information.

Supporting information.

Supporting information.

Supporting information.

Supporting information.

## Data Availability

The data that support the findings of this study are available from the corresponding author upon reasonable request. Data are available upon written request to the Corresponding Author.
