# Supplementary material for: Safety and Efficacy of a Novel Sealant‐Based Vascular Closure Device Following Electrophysiology Procedures: ReliaSeal Trial
Source: J Cardiovasc Electrophysiol. 2025 Mar 17;36(5):1022–31. doi: 10.1111/jce.16623 (PMC13020526; doi:10.1111/jce.16623)
Supplement: Supplementary file 2 — Supporting information. [file JCE-36-1022-s005.docx]

**Supplemental Figures and Tables for** **Safety and Efficacy of a Novel Sealant-Based Vascular Closure Device Following Electrophysiology Procedures: ReliaSeal Trial**

Supplemental Figure 1:

Supplemental Figure 1: Diagram showing Mynx Control Venous Vascular Closure Device and key features of the device.

Supplemental Video 1: Ultrasound image of Mynx Venous Vascular Closure Device in femoral vein. In the first clip, the vein is seen in cross section and the balloon at the tip of the device shaft is shown moving to vessel surface, which provides hemostasis and also facilitate sealant deposit on the venotomy surface and not within the vessel. In the second clip, the device is being retracted to trigger the deployment mechanism to allow sealant deposition. This clip shows tenting of the vessel and surrounding tissue indicating that the balloon remains in the vessel as manual traction is applied.

Supplemental Figure 2:

Supplemental Figure 2 Legend: Diagram illustrating the steps of Mynx Control Venous VCD Deployment

Supplemental Figure 3: Access Sites Per Limb

Supplemental Figure 3 Legend: Percentage of access sites per limb in the intervention arm (left graph) and control group arm (right graph). Over 1/3 of subjects had access sites in both limbs.

Supplemental Figure 4: Multiple Access Sites Breakdown

Supplemental Figure 4 Legend: The majority of subjects had two, three or four access sites, the 3 access sites being the most common. The majority of sheaths were 8.5 Fr or smaller sheaths but over 28% of sheaths were equal to or larger than 9Fr in size.

Supplemental Table 1: Distribution and Frequency of Procedure Types in the ReliaSeal Study

| Procedure type | MCV VCD (N=175) | Manual compression (N=91) |
| --- | --- | --- |
| AF ablation (or similar) | 67 | 29 |
| Ablation | 57 | 28 |
| SVT (or similar) | 11 | 8 |
| Right/left heart catheterization | 12 | 6 |
| AFL ablation (or similar) | 8 | 7 |
| Venogram | 7 | 3 |
| AVNRT ablation (or similar) | 4 | 2 |
| Other | 9 | 8 |

Supplemental Table 2: Distribution of Sheath Sizes Used in the ReliaSeal Study

| Guide catheter sheath size (Fr) | MCV VCD (N=177 patients, 470 access sites) | Manual compression (N=93 patients, 249 access sites) |
| --- | --- | --- |
| 6 | 7.2% (34/470) | 7.6% (19/249) |
| 6.5 | 0.4% (2/470) | 1.6% (4/249) |
| 7 | 16.8% (79/470) | 16.9% (42/249) |
| 7.5 | 0.9% (4/470) | 2.0% (5/249) |
| 8 | 42.1% (198/470) | 37.8% (94/249) |
| 8.5 | 4.0% (19/470) | 8.0% (20/249) |
| 9 | 20.2% (95/470) | 17.3% (43/249) |
| 9.5 | 0.0% (0/470) | 0.0% (0/249) |
| 10 | 4.9% (23/470) | 6.0% (15/249) |
| 10.5 | 0.0% (0/470) | 0.0% (0/249) |
| 11 | 2.8% (13/470) | 2.8% (7/249) |
| 11.5 | 0.2% (1/470) | 0.0% (0/249) |
| 12 | 0.4% (2/470) | 0.0% (0/249) |

Supplemental Table 2 Legend: Sheath sizes use in the Reliaseal study in the intervention group versus the manual compression groups. (MCV VCD = Mynx Control Venous Vascular Closure Device)

Supplemental Table 3: Post Procedure Pain Assessment

| Pain Score | MCV VCD  (N=177 subjects) | Manual Compression (N=93 subjects) | All  (N=270 subjects) |
| --- | --- | --- | --- |
| 0 | 64.2% (111/173) | 59.6% (53/89) | 62.6% (164/262) |
| 1 | 17.9% (31/173) | 12.4% (1189) | 16.0% (42/262) |
| 2 | 9.2% (16/173) | 11.2% (10/89) | 9.9% (26/262) |
| 3 | 2.9% (5/173) | 1.1% (1/89) | 2.3% (6/262) |
| 4 | 2.3% (4/173) | 4.5% (4/89) | 3.1% (8/262) |
| 5 | 1.7% (3/173) | 5.6% (5/89) | 3.1% (8/262) |
| 6 | 0.0% (0/173) | 1.1% (1/89) | 0.4% (1/262) |
| 7 | 1.2% (2/173) | 2.2% (2/89) | 1.5% (4/262) |
| 8 | 0.6% (1/173) | 0.0% (0/89) | 0.4% (1/262) |
| 9 | 0.0% (0/173) | 0.0% (0/89) | 0.0% (0/262) |
| 10 | 0.0% (0.173) | 2.2% (2/89) | 0.8% (2/262) |

Supplemental Table 3 Legend: Venous access site related pain was assessed at time of discharge eligibility and measured by pain score from 0-10. (MCV VCD= Mynx Control Venous Vascular Closure Device)
