# Supplementary figures and images for: Safety and Efficacy of a Novel Sealant‐Based Vascular Closure Device Following Electrophysiology Procedures: ReliaSeal Trial
Source: J Cardiovasc Electrophysiol. 2025 Mar 17;36(5):1022–31. doi: 10.1111/jce.16623 (PMC13020526; doi:10.1111/jce.16623)

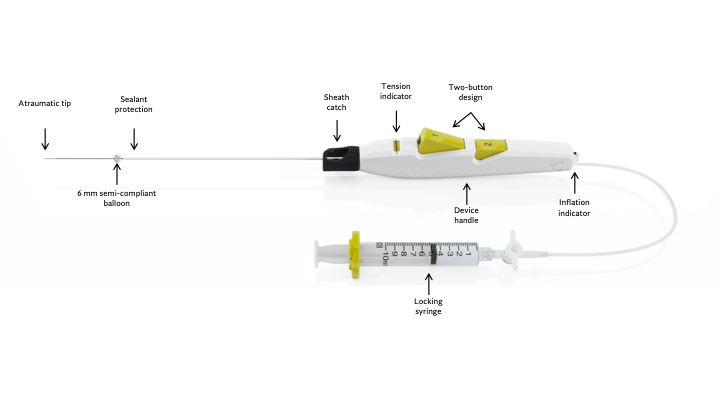

Supplement: Supplementary file 3 — Supporting information. [file JCE-36-1022-s003.tiff]

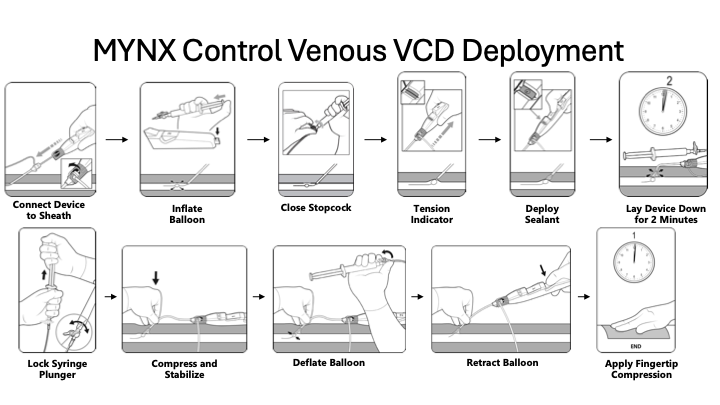

Supplement: Supplementary file 4 — Supporting information. [file JCE-36-1022-s002.tiff]

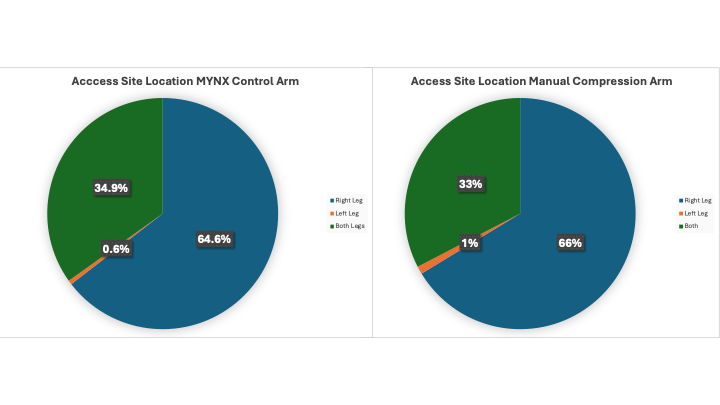

Supplement: Supplementary file 5 — Supporting information. [file JCE-36-1022-s001.tiff]

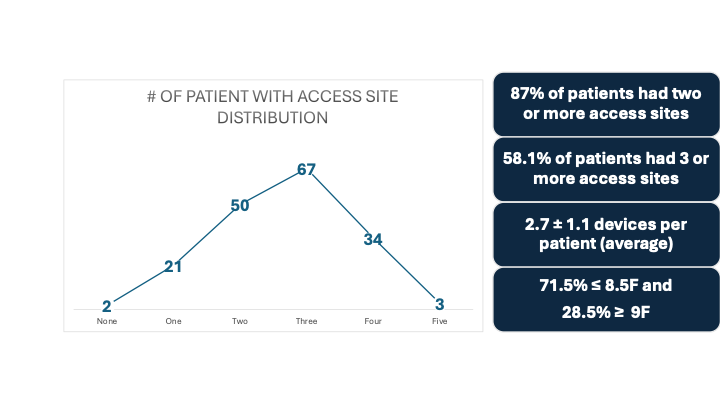

Supplement: Supplementary file 6 — Supporting information. [file JCE-36-1022-s004.tiff]
